# Redox potential tuning by calcium ions in a novel *c*-type cytochrome from an anammox organism

**DOI:** 10.1016/j.jbc.2024.108082

**Published:** 2024-12-13

**Authors:** Mohd. Akram, David Hauser, Andreas Dietl, Matthias Steigleder, G. Matthias Ullmann, Thomas R.M. Barends

**Affiliations:** 1 Department of Biomolecular Mechanisms, Max Planck Institute for Medical Research, Heidelberg, Germany; 2 Computational Biochemistry Group, Fakultät für Chemie, Biologie und Geowissenschaften, Bayreuth, Germany

**Keywords:** oxidation–reduction (redox), heme, electrochemistry, cytochrome *c*, electron transfer

## Abstract

The electrochemical potentials of redox-active proteins need to be tuned accurately to the correct values for proper biological function. Here, we describe a diheme cytochrome *c* with high heme redox potentials of about +350 mV, despite having a large overall negative charge, which typically reduces redox potentials. High-resolution crystal structures, spectroelectrochemical measurements, and high-end computational methods show how this is achieved: each heme iron has a calcium cation positioned next to it at a distance of only 6.9 Å, raising their redox potentials by several hundred millivolts through electrostatic interaction. We suggest that this has evolved to provide the protein with a high redox potential despite its large negative surface charge, which it likely requires for interactions with redox partners.

The redox potentials encountered in heme proteins span a wide range. Strikingly, this is achieved with a limited diversity in cofactor chemistry; *c*-type cytochromes, for instance, all use heme *c* as their redox cofactor, yet are able to tune their redox potential to any value from roughly −400 to +400 mV (1, 2, 3). To achieve this, they combine the effects of iron coordination, (de)solvation, and the positioning of charged protein side chains, main-chain dipoles, and buried waters, as well as the distortion of heme planarity to preferentially stabilize one redox state over the other (1, 4, 5, 6). However, while a correct redox potential is undoubtedly a necessary condition for proper biological function, it is probably rarely a sufficient one. For instance, there is likely to be a requirement for one or more binding sites, with the correct affinity and specificity, for redox partners that must be fulfilled as well, along with any other conditions for correct functionality that must be met. Such additional requirements may be at odds with the required redox potential, prompting evolution to come up with interesting ways to resolve the conflict.

Here, we describe a protein where this is the case: the diheme *c*-type cytochrome Kustd1711, a protein whose redox potential appears at odds with another property of the protein—the overall electrostatic charge. Kustd1711 is a putative electron transfer protein encoded on the nitrite oxidoreductase (*nxr*) gene cluster of anaerobic ammonium-oxidizing (anammox) bacteria (7, 8, 9, 10, 11) and consists of two heme-containing domains and one additional domain. We find that although the protein has a strikingly strong negative charge, which tends to lower redox potentials, the hemes have high redox potentials of ∼350 mV *versus* standard hydrogen electrode (SHE). Using X-ray crystallography, theoretical calculations, and electrochemistry, we show that this apparent contradiction is resolved by the presence of specifically bound calcium ions positioned unusually close to the heme iron atoms, which raises the hemes’ redox potentials by several hundred millivolts through electrostatic interactions. Understanding how evolution combines various effects to tune redox potentials while maintaining other properties of a protein is not only important for our understanding of redox biology but also relevant to the design of novel redox-active proteins.

## Results

**Figure 1:**
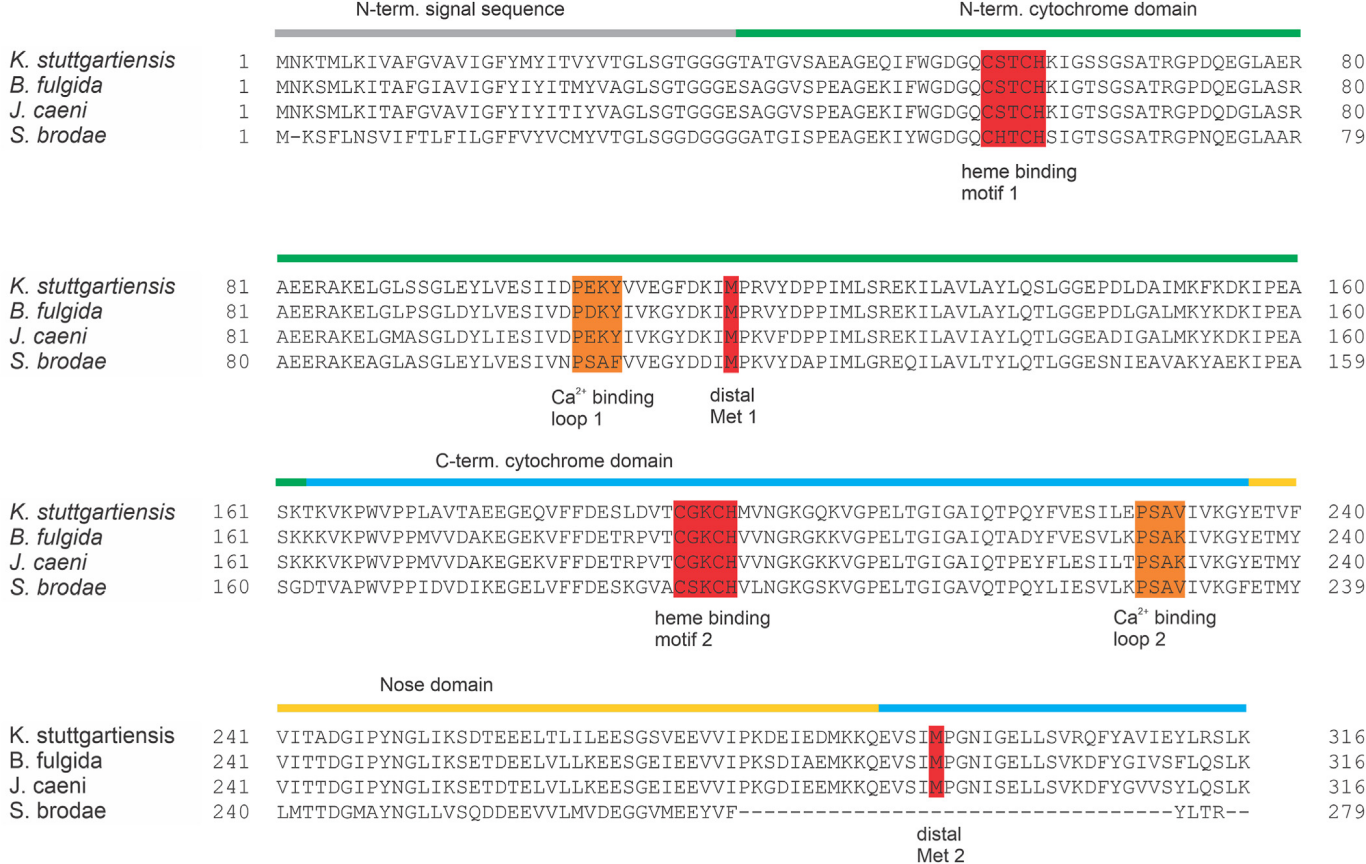
**Sequence features of Kustd1711.** Sequence alignment of Kustd1711 with its homologs from other anammox genera. The sequence entries shown are WP_099324718.1 (*Kuenenia stuttgartiensis*), KKO18758.1 (*Brocadia fulgida*), KAA0248123.1 (*Jettenia caeni*), and KHE93891.1 (*Scalindua brodae*), aligned with ClustalOmega (52). The domains and relevant residues are color coded and labeled.

**Figure 2:**
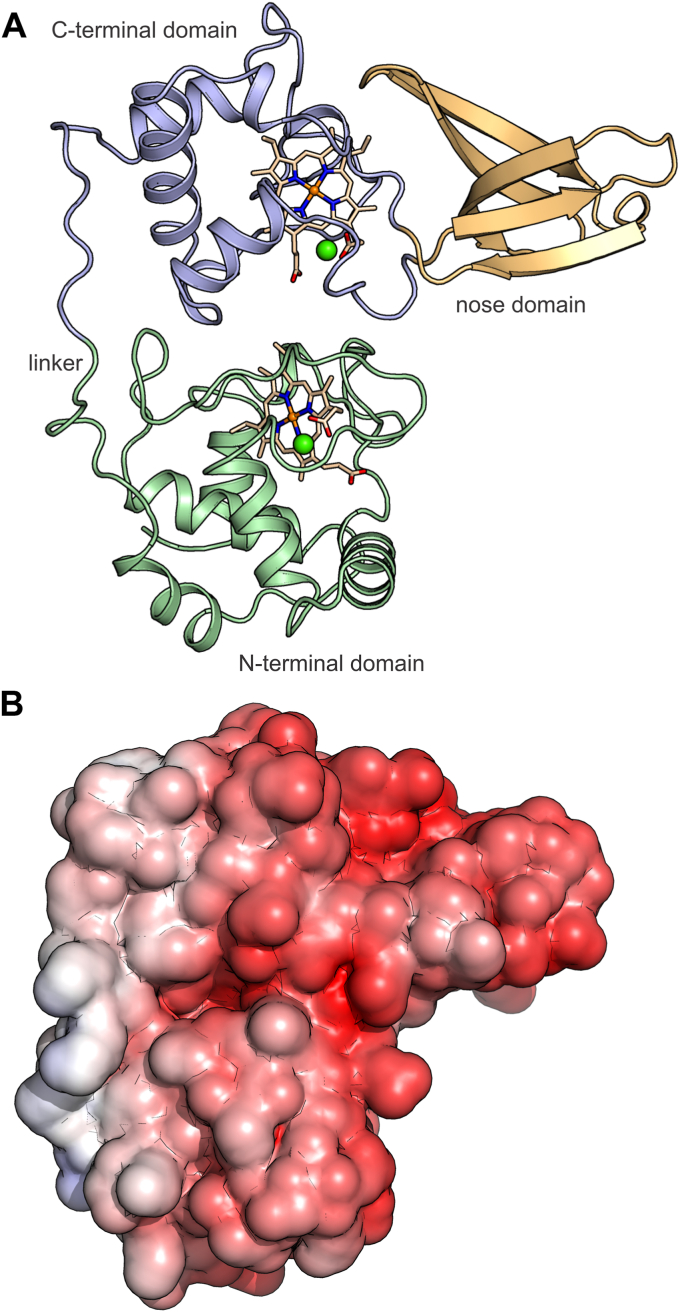
**Overview of the Kustd1711 structure.***A*, the N-terminal cytochrome domain is shown in *green*, the C-terminal cytochrome domain in *blue*, and the nose domain in *orange*. The heme groups are shown as *sticks*, and the calcium ions as *green spheres*. *B*, electrostatic surface properties as calculated by APBS (35). The electrostatic potential is shown on the solvent-accessible surface, colored from *blue* (+10 k_B_T/e) to *red* (−10 k_B_T/e) and was calculated while including the heme groups and calcium ions.

The sequence of Kustd1711 is highly conserved across anammox genera and indicates the presence of two heme *c* binding motifs. Moreover, an N-terminal signal sequence was detected indicating transport into the anammoxosome, the energy-conserving organelle of anammox organisms (12) (Fig. 1). The 1.75 Å resolution crystal structure of heterologously produced Kustd1711 determined after removal of the His tag reveals a three-domain protein consisting of an N-terminal and a C-terminal domain sharing a highly similar fold, as well as a “nose domain” consisting of β-strands that is an insertion on the C-terminal domain (Figs. 1 and 2*A*, Tables 1 and 2). The N- and C-terminal domains, which are connected by a long linker, both show a typical cytochrome *c* fold, and each bind a *c*-type heme, with an edge-to-edge distance between the hemes of the two domains of 12.9 Å. In our 1.75 Å resolution crystal structure, a glycerol molecule from the cryoprotectant binds in between the two hemes. We therefore collected data from a crystal cryoprotected in PEG 1000, which showed that without glycerol, the space between the two hemes is occupied by the side chain of Arg70, but that the structure was otherwise unchanged. As the data from this crystal were of lower quality, we performed all analyses using the 1.75 Å resolution data from the crystal cryoprotected with glycerol. Importantly, in our theoretical calculations of redox potentials (see later), the presence or absence of the glycerol or the Arg70 side chain between the heme groups did not affect the results by more than a few millivolts.

**Table 1:** Crystallographic data collection statistics

Dataset	wt peak	wt infl.pt.	wt remote	wt, no glycerol	M292C	M292H
Diffraction source	SLS PX-II	SLS PX-II	SLS PX-II	ESRF ID23–1	SLS PX-II	SLS PX-II
Wavelength (Å)	1.74010	1.74047	1.71603	0.88560	0.99988	0.99988
Temperature (K)	100	100	100	100	100	100
Detector	Pilatus 6M	Pilatus 6M	Pilatus 6M	EIGER16M	Pilatus 6M	Pilatus 6M
Crystal-detector distance (mm)	160	160	160	212.12	240	150
Rotation range per image (°)	0.15	0.15	0.15	0.1	0.2	0.2
Total rotation range (°)	120	120	120	120	110	2 × 300
Exposure time per image (s)	0.1	0.1	0.1	0.1	0.2	0.2
Space group	*P*2_1_2_1_2	*P*2_1_2_1_2	*P*2_1_2_1_2	*P*2_1_2_1_2	*P*2_1_2_1_2	*P*2_1_2_1_2
*a*, *b*, *c* (Å)	61.9, 92.3, 55.0	61.9, 92.3, 55.0	61.9, 92.3, 55.0	62.6, 92.1, 55.3	61.2, 92.2, 55.5	62.7, 92.7, 55.2
α, β, γ (°)	90, 90, 90	90, 90, 90	90, 90, 90	90, 90, 90	90, 90, 90	90, 90, 90
Mosaicity (°)	0.25	0.25	0.25	0.15	0.15	0.10
Resolution range (Å)	50–1.75 (1.80–1.75)	50–1.75 (1.80–1.75)	50–1.75 (1.80–1.75)	50–1.70 (1.75–1.70)	50–1.70 (1.74–1.70)	50–1.12 (1.15–1.12)
Total no. of reflections	580,480 (1616)	611,421 (1625)	317,225 (5103)	156,820 (10,890)	141,868 (10,213)	903,381 (8807)
No. of unique reflections	55,885 (991)	55,848 (990)	58,141 (2755)	63,858 (4955)	35,153 (2567)	211,675 (4298)
Completeness (%)	91.7 (21.9)	91.6 (21.9)	95.4 (60.9)	94.7 (93.5)	99.5 (99.8)	88.6 (24.2)
Redundancy	10.4 (1.6)	10.9 (1.6)	5.5 (1.9)	2.5 (2.3)	4.0 (4.0)	4.3 (2.1)
<*I*/σ(*I*)>	25.5 (1.2)	18.1 (0.8)	20.3 (1.3)	5.5 (1.4)	14.0 (1.5)	15.4 (1.2)
*R*_r.i.m._	0.075 (0.539)	0.124 (0.553)	0.049 (0.626)	0.127 (0.818)	0.047 (1.057)	0.042 (0.892)
CC_1/2_	1.000 (0.766)	0.999 (0.729)	0.999 (0.709)	0.984 (0.595)	0.999 (0.621)	0.999 (0.490)
Wilson *B* (Å^2^)	26.4	25.4	24.9	33.4	38.2	18.8

Values for the outer shell are given in *parentheses*.

<I/σ(*I*)> falls below 2.0 at 1.85 Å resolution for the inflection points dataset and at 1.80 Å resolution for the peak- and inflection point datasets. For the M292C dataset, it falls below 2.0 at 1.74 Å resolution and for the M292H dataset at 1.15 Å resolution. Statistics for the M292H dataset were calculated treating Friedel mates as independent reflections.

**Table 2:** Refinement statistics

Structure PDB entry	wt 7ZS0	wt, no glycerol 9FBK	M292C 7ZS1	M292H 7ZS2
Resolution range (Å)	50–1.75 (1.80–1.75)	50–1.75 (1.80–1.75)	50–1.70 (1.74–1.70)	50–1.12 (1.15–1.12)
Completeness (%)	95.4 (60.9)	94.7 (93.5)	99.5 (99.8)	88.6 (24.2)
σ cutoff	1.34	1.33	1.34	1.34
No. of reflections, working set	54,339^a^	34,601	33,350	201,004
No. of reflections, test set	3760	2221	1755	10,582
Final *R*_cryst_	0.169	0.194	0.197	0.124
Final *R*_free_	0.196	0.232	0.225	0.139
Cruickshank diffraction precision index	0.2	0.26	0.2	0.1
No. of atoms				
Protein	2133	2152	2156	4543^b^
Ca^2+^ ions	3	5	3	4
Heme atoms	86	86	86	146
Water	256	355	171	606
Total	2478	2598	2416	5299
RMSD				
Bonds (Å)	0.009	0.010	0.008	0.021
Angles (°)	0.995	1.110	0.951	1.725
Average *B* factors (Å^2^)				
Protein	28.5	29.17	35.9	21.0
Ca^2+^ ions	24.3	27.6	29.4	15.2
Heme atoms	20.8	20.55	26.4	13.7
Water	37.7	37.1	43.5	34.4
Ramachandran plot				
Most favored (%)	98.9	98.5	98.9	99.3
Allowed (%)	1.1	1.5	1.1	0.7
Outliers (%)	0.0	0.0	0.0	0.0

Values for the outer shell are given in *parentheses*.

a Treating Friedel mates as individual reflections.

b Including hydrogen atoms.

**Figure 3:**
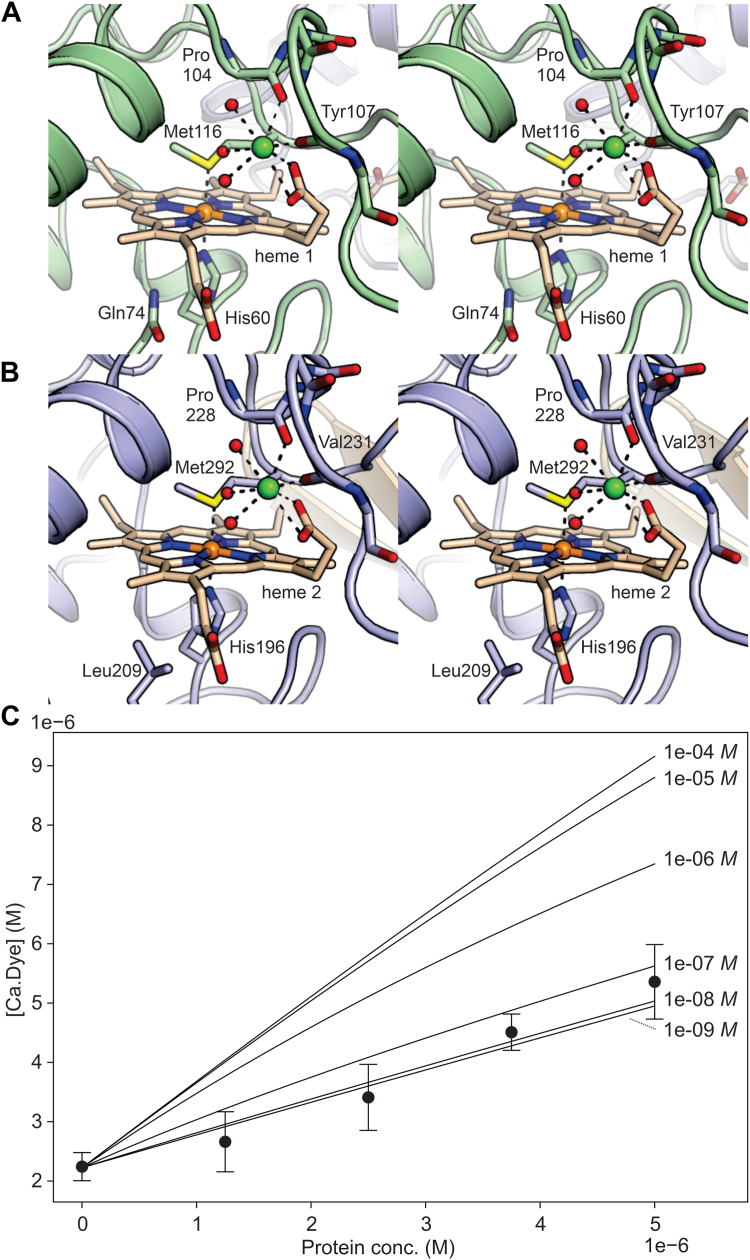
**Calcium- and heme-binding sites.***A*, heme in the N-terminal domain and (*B*) the C-terminal domain of Kustd1711. The main chain of the loop providing the calcium-coordinating carbonyl groups, the side chains of the heme-coordinating histidine and methionine residues and the heme groups themselves are shown as *sticks*, water molecules as *red spheres*, and the calcium ions as *green spheres*. The cysteine residues from the heme-binding CXXCH motifs have been omitted for clarity. *C*, determination of the apparent *K*_*d*_ of the protein–calcium complex by competition with the calcium-sensitive fluorescent dye CalBryte-590. The observed Ca-dye concentration as determined from the fluorescent signal is plotted as a function of protein concentration (*black circles*) together with the calculated curves for a range of *K*_*d*_ values. The error bars are standard deviations from experiments performed in triplicate.

The two heme-binding domains can be superimposed onto each other with a positional RMSD of 1.2 Å for 88 aligned Cα atoms (indeed, the two domains display ∼40% sequence identity). The most closely related structure to these two individual domains as found by the DALI server (13, 14, 15) is the SoxX cytochrome from *Rhodovulum sulfidophilum* (16). The hemes in both cytochrome *c* domains are His/Met coordinated (Fig. 3, *A* and *B*). The conformations of the two hemes are identical to within experimental error; when the two hemes of the wt structure are superimposed, the RMSD for all heme atoms is 0.09 Å. When analyzed by the NSD server (17, 18), the hemes show a practically identical distortion from perfect planarity, which can be described as mainly consisting of a combination of ruffling (*B1u*, ∼0.4 Å) and waving (*Eg(x)*, ∼0.2 Å) (17). A search of the PyDISH database of heme geometries (19) (https://pydish.bio.info.hiroshima-cu.ac.jp/) shows that these values are typical for His/Met-coordinated hemes in electron transfer proteins (using data from 570 entries of 3.0 Å resolution or better).

The heme binding sites are extremely similar as well. The only notable difference is the side chain of a single residue positioned next to the heme-coordinating His, which is Gln74 in the N-terminal domain and Leu209 in the C-terminal domain (Figs. 1 and 3, *A* and *B*). Importantly, both cytochrome *c* domains bind a metal ion in an identical manner, through interactions with main-chain carbonyl oxygens as well as interactions with one of the heme’s propionate groups. Superposition of the two hemes also brings these associated ions into virtually the same position (within 0.12 Å). These ions are further coordinated by three solvent molecules. The binding mode of these ions indicates that they are Ca^2+^ ions, which were present during crystallization, and modeling these ions as such explains the electron density excellently. Moreover, inductively coupled plasma optical emission spectroscopy (ICP-OES) elemental analysis of a typical preparation of heterologously produced His-tagged protein in the as-isolated state reveals the presence of ∼0.8 Ca atom per heme group (Table 3), which suggests that the presence of these ions is not a crystallization artifact. Indeed, a competition assay with the calcium-sensitive fluorescent dye CalBryte-590 allowed an upper limit of 1 μM to be established for the apparent dissociation constant (*K*_*d*_) for calcium in the as-isolated state of the protein (Fig. 3*C*). However, the *K*_*d*_ is likely to be much lower, as the fact that in the as-isolated state of the preparation used for elemental analysis, a considerable fraction of binding sites is loaded with Ca^2+^, despite the low-calcium conditions prevalent inside a typical bacterial cell.

**Table 3:** ICP-OES elemental analysis results

Sample	Ba (**μ**M)	Ca (**μ**M)	Cu (**μ**M)	Fe (**μ**M)	K (**μ**M)	Na (**μ**M)	Ni (**μ**M)	Sr (**μ**M)	Zn (**μ**M)
as-iso-1	0.12	556.39	3.89	691.17	53.71	6312.38	24.02	1.09	100.05
as-iso-2	0.12	558.88	3.84	696.55	53.45	6337.33	24.02	1.09	100.35
as-iso-3	ND	337.82	2.24	411.84	29.15	5189.62	14.24	0.66	58.13
EGTA-1	0.17	18.46	0.94	533.60	21.35	6287.43	ND	0.26	10.48
EGTA-2	0.17	17.71	0.92	526.44	21.48	6287.43	0.86	0.26	10.31
EGTA-3	0.17	17.96	ND	558.67	19.39	4191.62	ND	0.26	10.00

Sample	Ba (/Fe)	Ca (/Fe)	Cu (/Fe)	Fe (/Fe)	K (/Fe)	Na (/Fe)	Ni (/Fe)	Sr (/Fe)	Zn (/Fe)
as-iso-1	0.00	**0.80**	0.01	**1.00**	0.08	9.13	0.03	0.00	0.14
as-iso-2	0.00	**0.80**	0.01	**1.00**	0.08	9.10	0.03	0.00	0.14
as-iso-3	ND	**0.82**	0.01	**1.00**	0.07	12.60	0.03	0.00	0.14
EGTA-1	0.00	**0.035**	0.0018	**1.00**	0.04	11.8	ND	0.00	0.02
EGTA-2	0.00	**0.034**	0.0017	**1.00**	0.04	11.9	0.001	0.00	0.02
EGTA-3	0.00	**0.032**	ND	**1.00**	0.03	7.55	ND	0.00	0.02

Three samples each were investigated of as-isolated and EGTA-treated His-tagged protein; the *top half* of the table shows the results in absolute concentrations; the *bottom half* is normalized to the iron concentration. Assuming all the iron stems from the heme *c* moieties, there are approximately 0.8 calcium atoms per heme group in the as-isolated protein, whereas in the EGTA-treated protein, the amount of calcium is an order of magnitude lower.

Bold values indicate the amount of Ca and Fe.

ND, not determined.

The distance between each heme iron atom and its bound calcium is only 6.9 Å in each domain. To investigate how often a calcium ion is found this close to a heme iron atom, we mined the Protein Data Bank (PDB) for calcium-containing heme proteins. We found 561 structures containing one or more calcium ions as well as one or more heme moieties and extracted all 11,543 distances between calcium- and heme iron atoms. This showed that the distance observed here is the smallest reported to date; the next closest distance is 8.6 Å in the *Desulfovibrio gigas* cytochrome *c*3 (20). A list of all PDB entries investigated, and the distances extracted are given as Supporting information.

The presence of the “nose” domain insertion on the side of the C-terminal domain is thus the only major difference between the two cytochrome *c* domains. The nose domain consists of five β-strands divided into two “leaves” with one strand shared between them. The fold of this domain is highly similar to that of the bacterial RNA-binding Hfq proteins (21). However, while Hfq and related proteins show a highly positive surface charge as expected for nucleic acid–binding proteins, the reverse is true for the nose domain and indeed the entire Kustd1711 protein, which contains 48 glutamate and aspartate residues, but only 27 lysines and arginines, imparting a pronounced negative electrostatic potential to almost the entire surface of the protein (Fig. 2*B*).

**Figure 4:**
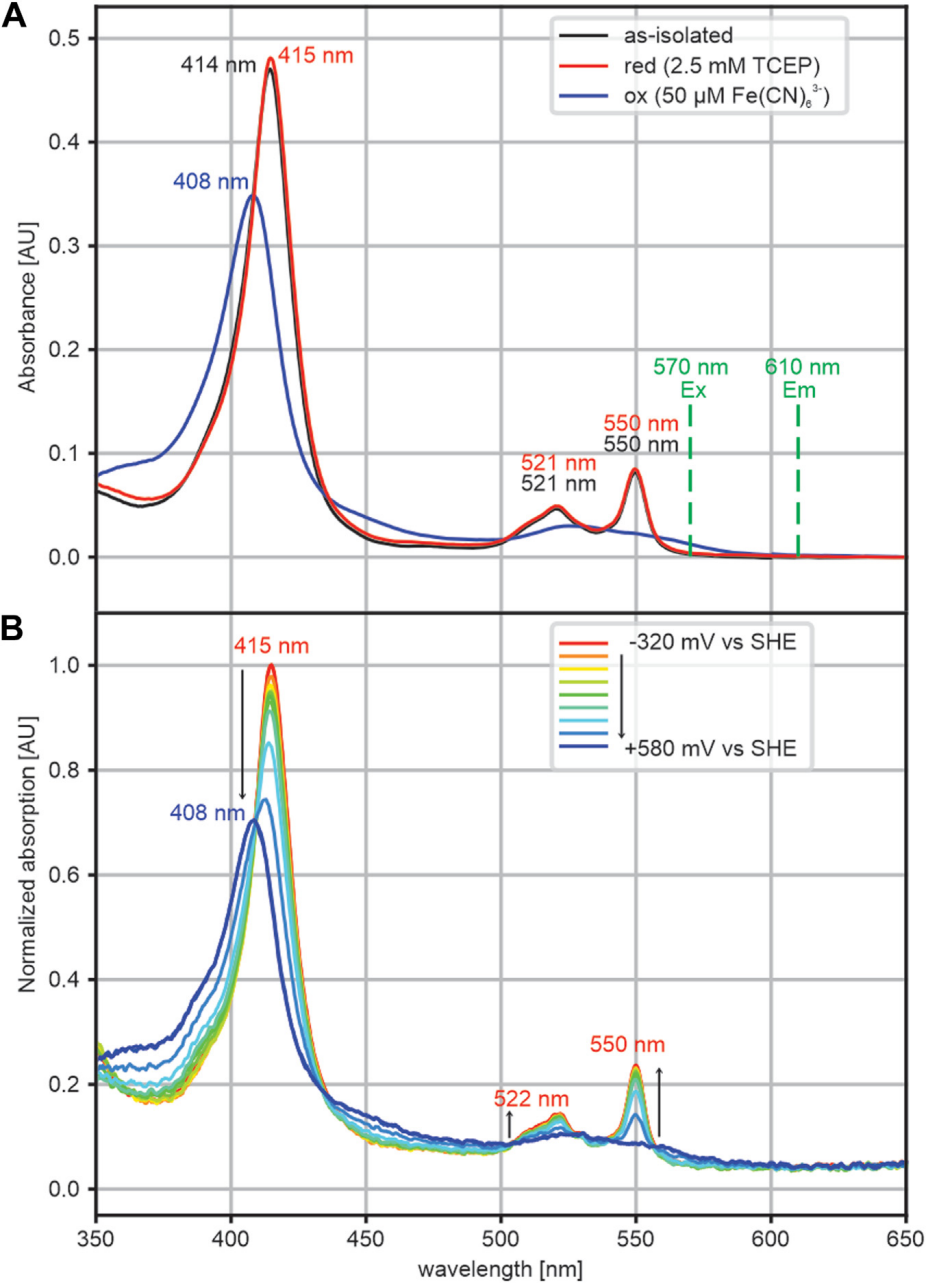
**UV–Vis spectroscopic features of Kustd1711.***A*, UV–Vis spectra of Kustd1711 stored without reductive agents measured in a cuvette (*black*), reduced with 2.5 mM TCEP (*red*), and oxidized with 50 μM potassium ferricyanide (*blue*). The *green lines* indicate the excitation and emission wavelengths used in the fluorometric calcium affinity determination. *B*, family of UV–Vis spectra of Kustd1711 collected in an OTTLE cell during a spectroelectrochemical titration. The spectra are colored from *red* (−320 mV) to *blue* (+580 mV). Note that in *B*, the spectra are normalized to the absorption maximum observed at −320 mV *versus* SHE. OTTLE, optically transparent thin-layer electrode; SHE, standard hydrogen electrode; TCEP, Tris(2-carboxyethyl)phosphine hydrochloride.

**Figure 5:**
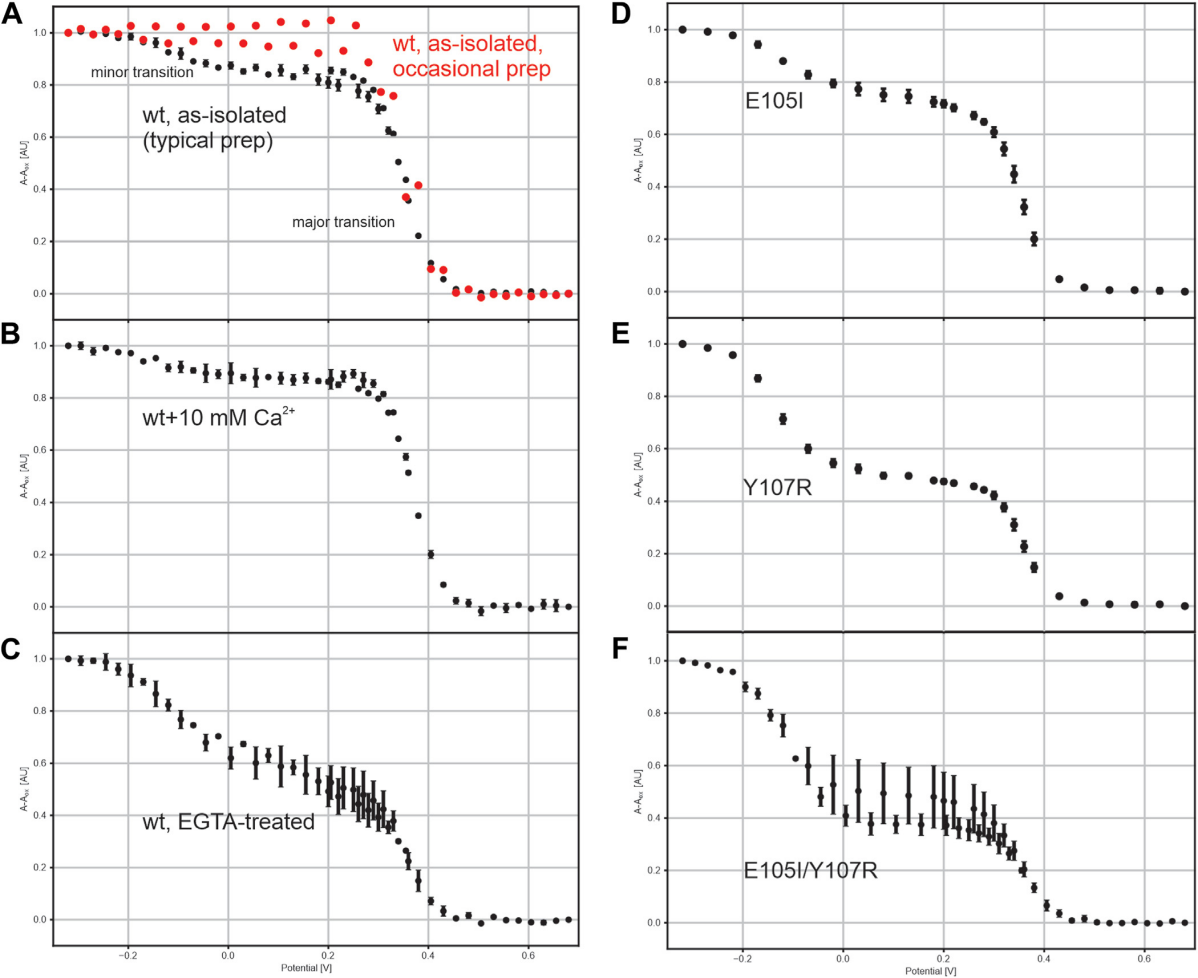
**Properties of wt and mutant Kustd1711.***A*–*F*, spectropotentiometric traces for wt, as-isolated protein (*A*), in the presence of 10 mM Ca^2+^ (*B*), and after treatment with EGTA (*C*). A major and a minor transition is observed in the data from typical preps the as-isolated protein at high and low potential, respectively (*black data*). Occasionally, a prep of the wt protein only shows the high-potential transition (*red data* in *A*). The relative amplitude of the minor transition is slightly decreased in the data from Ca^2+^-treated protein, whereas it is strongly increased in the data from protein treated with EGTA. In the calcium-binding loop mutants E105I (*D*), Y107R (*E*), and E105I/Y107R (*F*), the relative amplitude of the minor transition is increased. For the single mutants, only the oxidative branch of the measurement is shown. All traces were measured in triplicate, and the error bars are standard deviations. The potential (*x*-axis) is indicated in volts *versus* standard hydrogen electrode.

As the sequence and structure suggest a role in electron transfer, we investigated the redox properties of the protein. We first measured UV–Vis spectra in the as-isolated state as well as after treatment with reducing- and oxidizing agents (Fig. 4*A*). Note that a strong reducing agent (Tris(2-carboxyethyl)phosphine hydrochloride [TCEP]) was present during isolation, which was, however, removed in later stages and not present in the storage buffer. In the as-isolated state, the protein showed a strong Soret absorption at 414 nm accompanied by clear α- and β bands at 550 and 521 nm, respectively. Treatment with 2.5 mM of the reductant TCEP slightly shifted the Soret band to 415 nm but did not significantly affect the intensities of either the Soret absorption or of the α- and β bands. Oxidation of the protein with 50 μM of the oxidizer potassium ferricyanide shifted the Soret band to 408 nm and decreased its intensity, while flattening out the α- and β bands. We next performed a spectropotentiometric characterization of the protein, monitoring the change in absorption at 417 nm (where the largest absorption change occurs upon oxidation) at different preset potentials in an optically transparent thin-layer electrode (OTTLE) cell (Figs. 4*B* and 5). For most preparations of the wt protein, this revealed two redox transitions for the wt protein in the as-isolated state after removal of the His tag (Fig. 5*A*, *black data points*): a major and high potential transition with an amplitude of typically ∼85% of the total signal, centered at +353 mV, and a minor (typically ∼15% of the total signal), broad transition at low potential, centered at −92 mV *versus* SHE. The relative sizes of these transitions were not constant between preparations and conditions; in some cases, the minor, low-potential transition could not be observed (Fig. 5*A*, *red data points*). Starting from the typical preparation displaying low- and high-potential transitions shown in Figure 5*A* (*black data points*), inclusion of 10 mM Ca^2+^ resulted in the major transition being at 370 mV at a relative amplitude of 89%, whereas the minor transition had decreased to 11% relative amplitude and was located at −127 mV *versus* SHE (Fig. 5*B*). When this same preparation was treated with EGTA in an attempt to remove Ca^2+^, the major transition decreased in relative amplitude as well as in potential, which moved to +344 mV. The minor transition was much less well defined in this case but appeared to have a much larger relative amplitude and was centered at approximately −78 mV *versus* SHE (Fig. 5*C*).

At first glance, the observation of two redox transitions for a protein with two heme groups seems trivial to explain, by assuming each transition is caused by one of the hemes. However, the fact that the minor transition was not observed for every preparation of the protein already makes this very unlikely. Moreover, the large difference in both midpoint potential and amplitude between the two transitions, despite the large degree of similarity between the heme sites, makes such a scenario even more unlikely. Thus, to explain the observed redox behavior, we performed continuum electrostatic calculations (22) of the redox potentials of the two heme sites, starting from the crystal structure coordinates of the wt protein. The calculated redox potentials of the hemes in the N- and C-terminal domains turned out to be very similar, being +308 mV for the N-terminal heme and +296 mV for the C-terminal heme. While these calculated potentials are somewhat lower than the major, high potential transition observed in spectroelectrochemistry (Fig. 5, *A* and *B*), the small difference between the two calculated potentials does suggest that the high potential redox transition observed for the as-isolated and calcium-treated protein is due to two virtually superimposed transitions, one from each heme-binding domain, which cannot be distinguished with our spectropotentiometry setup. Moreover, calculations without calcium ions in the binding sites resulted in calculated redox potentials of −41 mV and −83 mV for N- and C-terminal domains, respectively. This is consistent with the position of the minor, low-potential transition observed electrochemically and suggests that this transition, too, corresponds to a superposition of two separate redox transitions. In line with this, the relative intensity of the high-potential transition decreases upon treatment with EGTA, whereas the low-potential transition becomes more pronounced, though much broader (Fig. 5*C*).

We next tried to modify the calcium binding of the site in the N-terminal domain through mutation. This, however, is complicated by the fact that the calcium only interacts with protein backbone carbonyl groups, the heme propionate and water, not with any side chains. Thus, we mutated two residues in the calcium-binding loop in an effort to change the backbone conformation. Glu105, located on the outside of the calcium-binding loop of the N-terminal domain, does not directly coordinate the calcium ion but forms a salt bridge with Arg118 in the wt protein and was mutated to Ile to preclude this interaction and change the loop's conformation to reduce calcium affinity. Indeed, in the Glu105Ile mutant, the low-potential transition increased in relative amplitude (Fig. 5, *D–F*). This effect was even stronger when Tyr107, whose carbonyl group coordinates the calcium ion and whose side chain also approaches Arg118, was mutated to Arg. In the double mutant E105I/Y107R, the effect was stronger still. These results are consistent with an influence of calcium binding on the redox potential, but it should be noted that these observations could also be at least partially explained by second-sphere effects on the heme potentials not involving the presence or absence of calcium.

**Figure 6:**
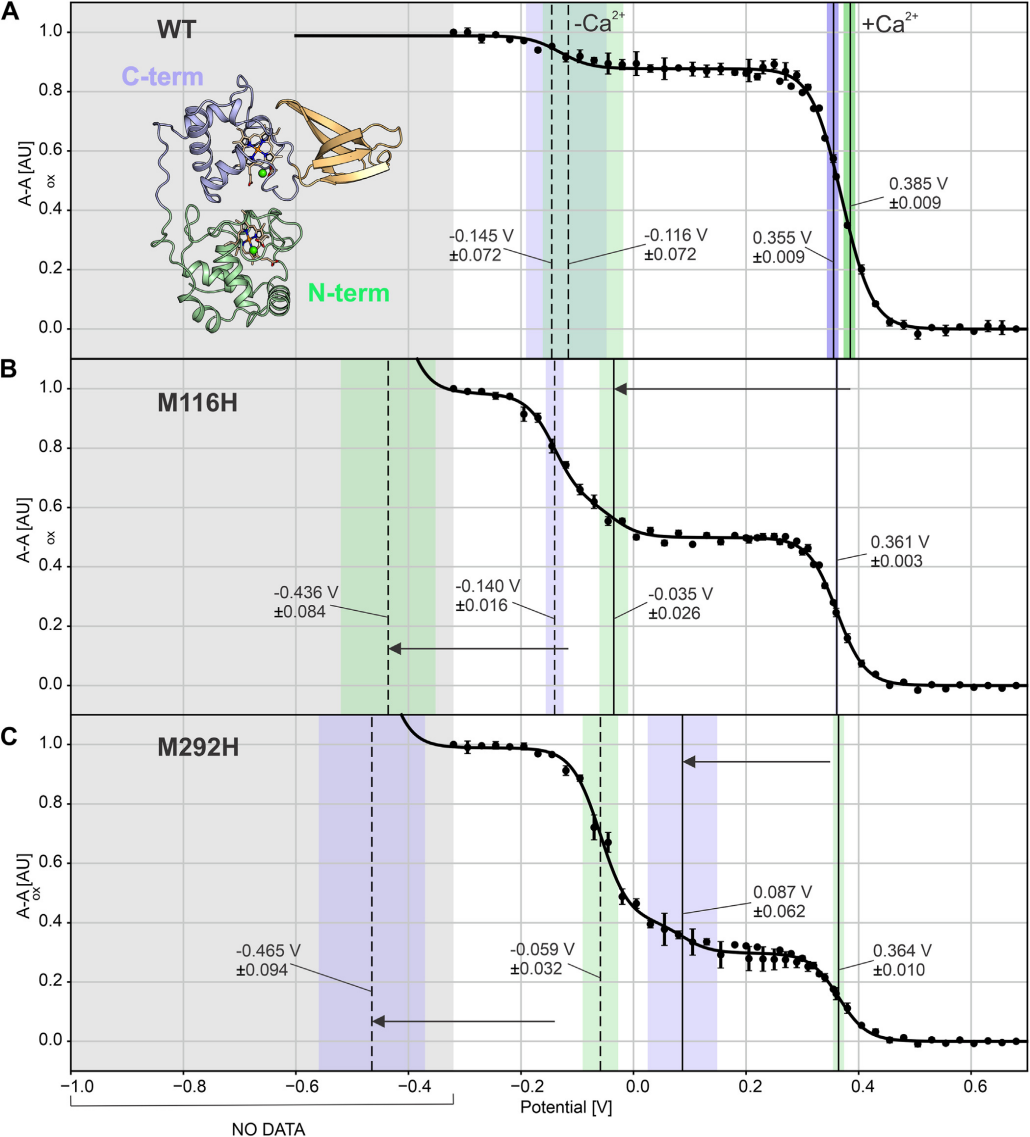
**Spectroelectrochemical fitting results.***A*, wt protein in the presence of 10 mM Ca^2+^. *B*, M116H mutant. *C*, M292H mutant. The *solid traces* are the fit results with four one-electron transitions as described in the text. The *vertical lines* indicate the potentials of the fitted transitions, and the *shaded areas* are the standard uncertainties of the fit results. The *green*- and *blue*-labeled transitions are interpreted as arising from the N- and C-terminal heme domains, respectively. A *solid vertical line* indicates a transition in a calcium-loaded heme site, and a *dashed vertical line* indicates in a calcium-free heme site. The potential (*x*-axis) is indicated in volts *versus* standard hydrogen electrode.

To further validate our model, we tried to explain the spectroelectrochemical results obtained in the presence of calcium by fitting a four-transition Nernstian function to datasets showing clear minor and major transitions. In the fitting procedure, we assumed four redox transitions comprised of two closely spaced pairs: one pair at high potential, for the Ca^2+^-loaded case, and one pair at lower potential, for the Ca^2+^-free case, with the same spacing in potential within each pair, and the same occupancy for the two members of each pair. This resulted in an excellent fit (Fig. 6*A*) with a potential difference of 500 mV between the two pairs and a spacing of 30 mV within each pair, with the individual transitions at −145, −116, +355, and +385 mV *versus* SHE. The difference in potential within each pair of 30 mV that was obtained by fitting is consistent with the small spacings between the calculated potentials in the respective calcium-bound and calcium-free states. The occupancy for each of the two individual low potential transitions refined to 6% and 44% for each of the two high-potential transitions, suggesting 88% occupancy of the calcium sites.

Taking into account that the calculations predicted the heme in the N-terminal domain to have a slightly higher potential than the C-terminal domain heme, one might tentatively assign the fitted redox potentials to the two hemes in their respective calcium-bound and calcium-free states as follows: −145 mV transition: C-terminal heme, no Ca^2+^/−116 mV: N-terminal heme, no Ca^2+^/+355 mV: C-terminal heme, with Ca^2+^/+385 mV: N-terminal heme, with Ca^2+^.

**Figure 7:**
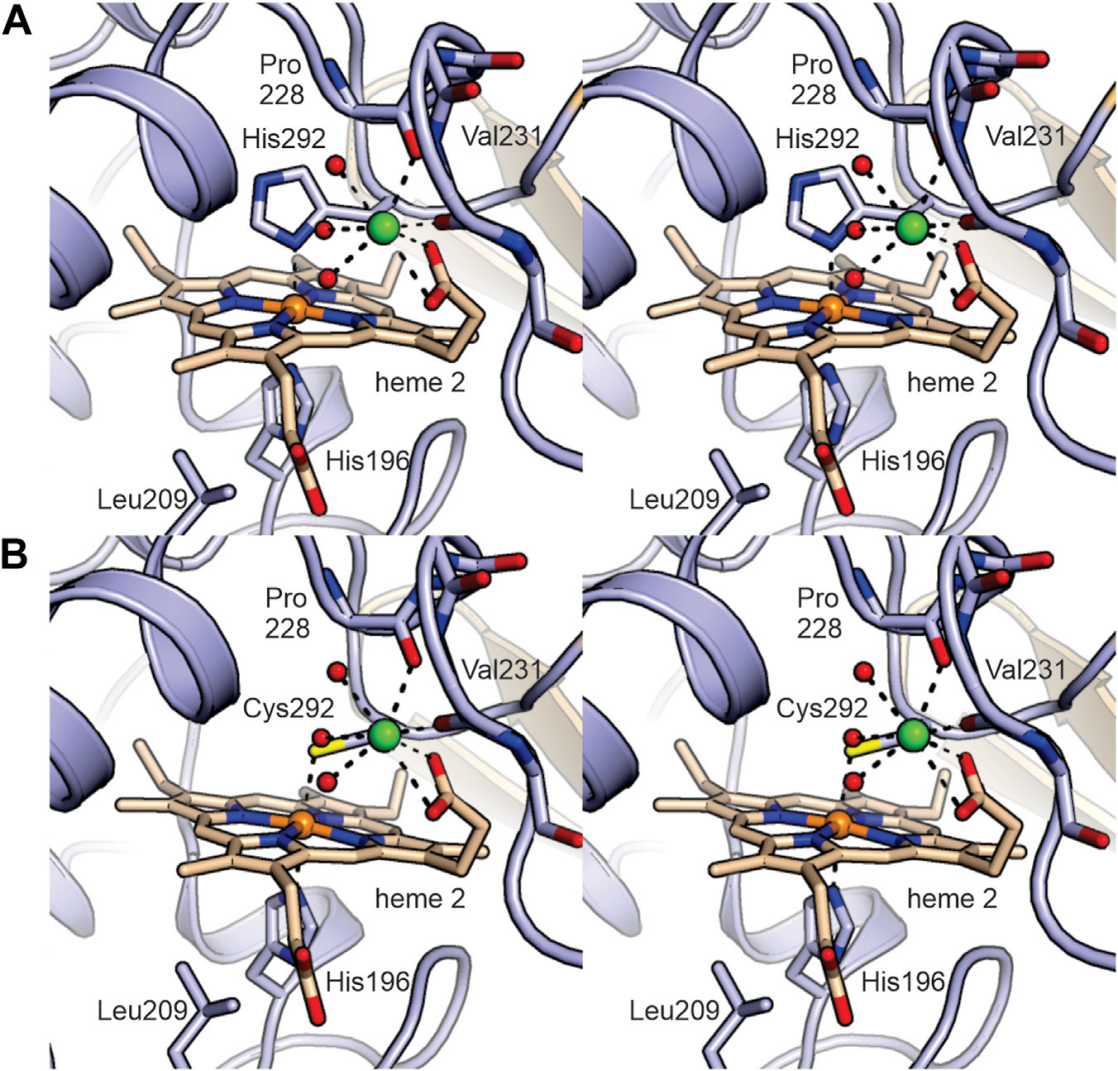
**N-terminal domain heme sites of KustD1711 mutants.***A*, M292H mutant and (*B*) M292C mutant of Kustd1711. Despite the mutation of the distal iron-coordinating residue, both heme coordination and the calcium-binding site are intact.

To improve our confidence in the assignment of the various transitions, we mutated the distal methionine ligands in both the N- and C-terminal heme sites to histidine and cysteine. To confirm that the sites can accommodate these mutations while maintaining six-coordinate heme binding and calcium binding, we determined the crystal structures of the C-terminal domain methionine mutants M292H and M292C to 1.12 and 1.70 Å resolution, respectively (Fig. 7, Tables 1 and 2) using the same crystallization conditions as for the wt protein, that is, including 200 mM Ca^2+^. This showed that these mutants are able to support both six-coordinate heme ligation as well as calcium binding, although in the M292H mutant, the plane of the distal His292’s side chain is at an angle of ∼75° with the plane of the heme porphyrin system rather than perpendicular to it. The corresponding mutants of the N-terminal heme site (M116C and M116H) did not yield crystals.

We then performed spectroelectrochemical measurements with these mutant proteins. The M→C mutants did not show clearly defined transitions at low potentials and showed considerable hysteresis, which precluded meaningful fitting (Fig. S2). The M→H mutants, however, yielded data with clear transitions and little hysteresis. As the mutation of a distal methionine to histidine in a *c*-type cytochrome typically results in a drop in redox potential of ∼320 mV (23), in each mutant, one of the two transitions in each of the two pairs can be expected to drop by approximately this amount. Thus, for both mutants, there should now be four clearly separated transitions: around +370 and −130 mV, for the unmutated domain in the calcium-loaded and calcium-free states, respectively, and around +50 and −450 mV, for the mutated domain in its calcium-loaded and calcium-free states, respectively. Thus, importantly, one of the transitions (for the calcium-free mutated heme domain, at around −450 mV) will fall outside the range of applied potentials used during our measurements, and another one (for the calcium-loaded mutated domain) will fall in between the high and low transitions observed in the wt protein, leaving three observable transitions in the potential range investigated. Indeed, the spectroelectrochemical traces obtained for the mutants can be explained excellently with three one-electron transitions in the expected potential ranges, and the result of fitting the traces accordingly is shown in Figure 6, *B* and *C*. This provides further support for our interpretation of the spectroelectrochemistry of the wt protein. Moreover, since the potentials assigned to the nonmutated heme sites are conserved in the data from the mutant proteins, particularly for the M116H mutant, the data appear to support the assignment of the various transitions to the N- or C-terminal domain hemes.

**Figure 8:**
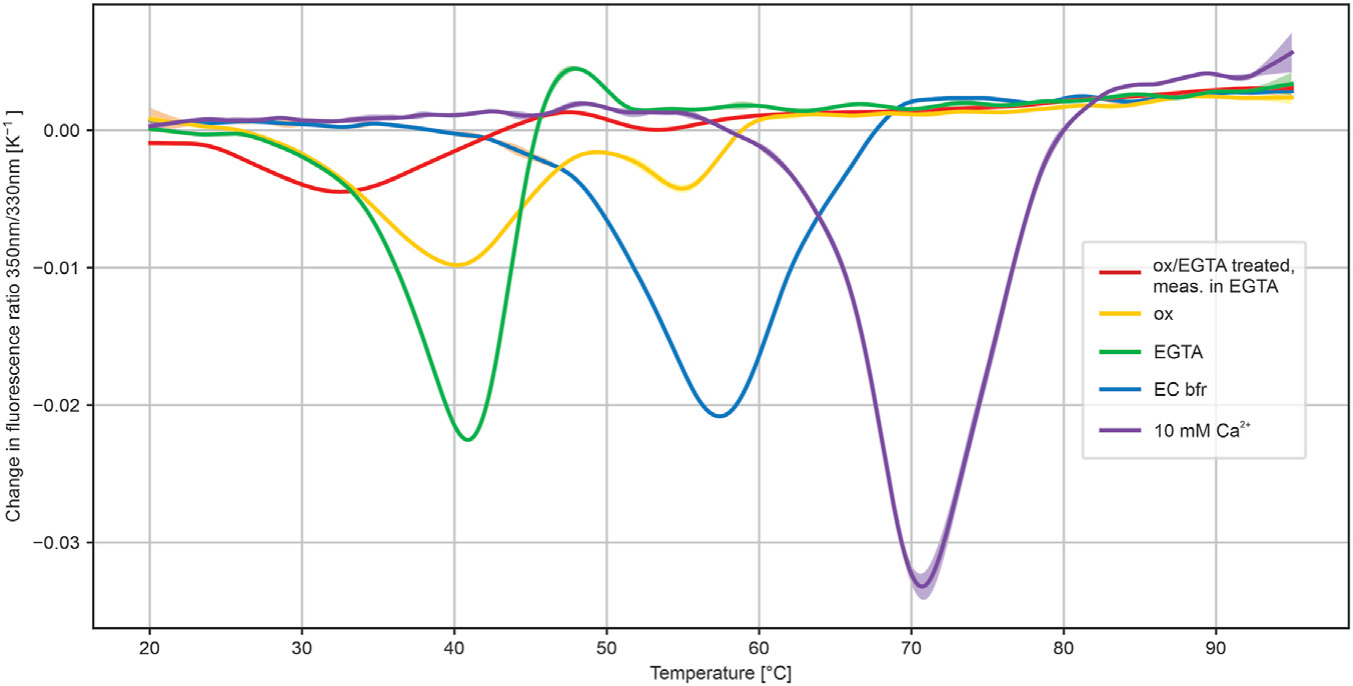
**Thermal stability of wt Kustd1711 in different conditions as determined by differential scanning fluorimetry.** Shown is the first derivative of the fluorescence ratio. *blue line*: EC buffer (100 mM KCl, 10 mM Mops–KOH [pH 7.0]); *purple line*: 10 mM calcium chloride in EC buffer; *green line*: 10 mM EGTA in EC buffer; *yellow line*: 10 mM potassium ferricyanide in EC buffer; *red line*: protein treated for 30 min on ice with 100 mM EGTA, 10 mM potassium ferricyanide in EC buffer, and then measured in 10 mM EGTA in EC buffer.

Finally, we performed differential scanning fluorimetry (DSF) to investigate the stability of the protein in various states (Fig. 8). The protein displayed the highest unfolding temperature (71 °C) in the presence of 10 mM Ca^2+^. In the buffer used for the spectropotentiometry experiments (10 mM Mops [pH 7.0] and 100 mM KCl), the protein unfolded at 57 °C. Treatment of the protein with EGTA reduced the unfolding temperature to 42 °C; however, an oxidation with ferricyanide resulted in an unfolding peak at a very similar temperature (40 °C), be it accompanied by a second smaller peak at 55 °C. Treatment with both EGTA and ferricyanide, that is, removal of the calcium as well as oxidation resulted in the lowest unfolding temperature, 32 °C.

## Discussion

Our results thus indicate that the major high-potential redox transition at +350 mV observed for as-isolated Kustd1711 can be attributed to two virtually superimposing redox transitions, one in each heme site in the calcium-bound state. Remarkably, despite the close proximity of the two heme groups in the crystal structure (as noted, the edge-to-edge distance is 12.9 Å), our calculations found no evidence for redox cooperativity. In fact, the effective interaction between the two heme centers is only about 0.2 kcal/mol. Thus, it appears Kustd1711 has evolved to have two heme domains that are electrochemically almost identical yet separate entities. The low-potential transition observed is consistent with a similar superposition of the redox transitions of the calcium-free states of the two heme sites. Moreover, the calculations, supported by mutational data, allow a tentative assignment of the individual transitions.

In addition, mutations of either of the heme sites’ distal heme-coordinating methionine to histidine change the relative amplitudes of the various transitions. To at least some extent, this could be explained by changes in extinction coefficients caused by the mutation. However, it is also possible that these mutations have compromised calcium binding. As the calcium-binding site is on the distal side of the heme (Fig. 3), and the loop containing the distal residue is adjacent to the calcium-binding loop, it is possible that small changes in the local structure have reduced the affinity for calcium ions in the mutated heme-binding domain, though at present this remains speculative as the determination of calcium affinities of the mutated sites is complicated by the presence of the remaining native binding site. That the crystal structures of the mutant showed a fully occupied calcium site in both domains is probably because of the high (200 mM) calcium concentration used in crystallization.

The presence of both the high- and low-potential transition in spectroelectrochemical data with EGTA-treated protein suggests the presence of calcium, although the ICP-OES data showed that EGTA treatment efficiently removes calcium from the protein (Table 3). Possibly, given the high affinity of the wt protein for calcium (apparent *K*_*d*_ <1 μM, see aforementioned), this calcium leached from the glass surface of the OTTLE cell. However, the spectroelectrochemical data also suggest that incubation with 10 mM calcium chloride did not result in full occupancy of the calcium-binding sites, despite the low apparent *K*_*d*_ for calcium in the as-isolated state of the protein. This apparent contradiction might be resolved if part of the protein is defective in calcium binding (at least on the timescale of the experiment), possibly by a binding-incompetent conformation of the calcium-binding loop, which is either unable or slow to reorganize into a binding-competent conformation. In particular, a slow switching between conformations could at least in part explain the hysteresis in some of our electrochemical experiments.

Importantly however, if calcium binding preferentially destabilizes the oxidized state of the heme over the reduced state, then the oxidation state of the heme must necessarily influence the calcium affinity. Such a coupling of the redox- and calcium-binding equilibria may affect the potential at which a redox transition is observed in bulk protein in a calcium-dependent manner. For instance, when the total calcium concentration is comparable to the dissociation constant, and the redox potential is close to the redox transition for the calcium-loaded case, the fraction of oxidized protein may differ from what would be expected from a single-transition Nernst equation alone, because oxidation of the protein changes the calcium-binding equilibrium favoring calcium release, which in turn favors oxidation. Thus, at such calcium concentrations, the redox transition will appear to occur at lower potentials than at saturating calcium concentrations; experimentally, one observes macroscopic redox transitions, which depend on the microscopic redox potentials as well as on the calcium concentration and affinity and the concentration of the protein. This might explain why the potential of the major transition observed for the wt protein was lowest (around +344 mV) in the EGTA-treated state, higher (+353 mV) in the as-isolated state, and highest (+370 mV) in the presence of 10 mM Ca^2+^ (Fig. 5, *A–C*). However, neither the Kustd1711 concentration nor the calcium concentration in the anammoxosome are currently known, and we were unable to determine the calcium affinity in the oxidized state because of interference of oxidizing agents with the fluorescent Ca-sensitive dye. Thus, the effective redox potential under physiological conditions cannot at present be estimated. However, DSF (Fig. 8) shows that while in the presence of 10 mM Ca^2+^, the unfolding temperature of the protein is >70 °C, treatment with EGTA and/or oxidation of the protein using ferricyanide (which favors calcium release) lowers the unfolding temperature of the protein dramatically. Indeed, when both EGTA treatment and oxidation by ferricyanide are applied, the unfolding temperature drops to a little over 30 °C, the temperature at which *Kuenenia stuttgartiensis* is usually cultured (24). Thus, it seems unlikely that large amounts of Kustd1711 would be in a calcium-free state in living *Kuenenia* cells, suggesting that the functional form of the protein is calcium loaded, and consequently has a high redox potential of around +350 mV.

Indeed, given its position in the genome on the *nxr* gene cluster encoding the NXR nitrite oxidoreductase (11), Kustd1711 likely performs a role in electron transfer within the cellular nitrite oxidation/nitrate reduction machinery, which uses electrons at a redox potential of roughly +400 mV. A high redox potential for Kustd1711 as observed in the calcium-loaded state is therefore not unexpected; indeed, the *nxr* gene cluster encodes a number of blue copper proteins, too (7), which are known for their high redox potentials. On the other hand, the protein has also evolved to have a pronounced negative surface charge. This likely serves to mediate interactions with other proteins, but the many negatively charged residues would stabilize the ferric state of the heme irons more than their ferrous state, which would lower their redox potentials. Our data and calculations show how this effect is counteracted: the presence of a divalent calcium ion at a distance of only 6.9 Å from each heme iron destabilizes the ferric state, resulting in the observed high redox potentials. Redox potential steering in this way has been suggested before; in a study of *D. gigas* cytochrome *c*3 in which theoretical calculations of the heme redox potentials were performed, inclusion of the calcium ions observed in the crystal structure was required to reproduce the redox potentials observed experimentally (25). The 6.9 Å distance between the calcium ions and the heme iron atoms in the present study is the shortest described so far, resulting in a large increase in redox potential. The results presented here expand our knowledge of the repertoire of structural features that nature can combine with each other to arrive at a protein with the correct combination of precisely tuned parameters required for biological function.

## Experimental procedures

### Protein expression and purification

wt and mutant Kustd1711 proteins were produced by heterologous expression in *Shewanella oneidensis* MR-1 (26) as described previously for another *c*-type cytochrome from *K. stuttgartiensis*, KsNaxLS (27). Briefly, the kustd1711 gene was cloned into a pUC19-derived vector (28); mutant constructs were prepared using the QuikChange method. The native N-terminal signal peptide was replaced by the strong signal peptide from the small tetraheme cytochrome *c* (SO2727) of the expression host *S. oneidensis* MR-1 (29). The pUC19-derived vectors containing the inserts were transformed into *S. oneidensis* MR-1 ΔendA (26) by electroporation at 0.55 kV, 25 μF, 200 Ω using a Gene Pulser II (Bio-Rad). For large-scale purification, 2 l batches (6–10 l total) of LB medium were supplemented with 50 μg/ml kanamycin and inoculated with 1% (v/v) of overnight culture of appropriately transformed *S. oneidensis* MR-1. Initially, large-scale cultures were grown in 5-L Erlenmeyer flasks (without baffles) at 30 °C while shaking at 100 rpm for 5 to 6 h, upon which the temperature was lowered to 20 °C, and the cultures were shaken at 60 rpm for a further 60 to 70 h. The cells were then harvested by centrifugation at 6000 rpm for 10 min at 4 °C in a Fiberlite F9-6 x1000 LEX rotor (Thermo Scientific). The cell pellets were either directly used for purification or frozen in liquid nitrogen and stored at −80 °C. The expressed proteins were purified using immobilized metal ion affinity chromatography using Ni–NTA agarose (Qiagen) followed by size-exclusion chromatography on a Superdex 75 (10/300 GL) column using an Äkta Purifier FPLC system (GE Healthcare) as described (27). For protein crystallography and electrochemistry, the hexahistidine tags were cleaved off by overnight incubation with tobacco etch virus protease in a buffer containing 150 mM NaCl, 20 mM Tris–HCl, pH 8.0, 2 mM TCEP prior to the size-exclusion step. His tags were not cleaved off for protein destined for elemental analysis (see later). The buffer was exchanged to 25 mM Hepes/NaOH, 25 mM KCl, and the protein concentrated to an $A_{550\;nm}^{1\;cm}$ of ∼21 (*i.e.*, where a 1:100 dilution has an $A_{550\;nm}^{1\;cm}$ of 0.21) by ultrafiltration in Amicon concentrators with a 10 kDa cutoff membrane.

### Structure determination and analysis

Crystals of untagged Kustd1711 were grown in hanging drop vapor diffusion setups, equilibrating drops of 1 μl of protein stock solution mixed with 1 μl of reservoir solution against 600 μl of reservoir, with the reservoir solution containing 0.2 M calcium acetate, 0.1 M imidazole/HCl (pH 8.0), and 20% (w/v) PEG 1K. Red crystals of several 100 μm in size grew within a few days and were cryoprotected in reservoir solution with 24% (w/v) glycerol prior to flash cooling in liquid nitrogen. A three-wavelength multiple-wavelength anomalous diffraction dataset was collected from a crystal of wt Kustd1711 at the PX-II beam line of the Swiss Light Source (Paul Scherrer Institute). Multiple-wavelength anomalous diffraction phasing using the anomalous signal of the heme irons, density modification, and the building of an initial model were performed automatically using PHENIX.autosolve (30, 31, 32): automatic substructure solution found two iron sites, which were used to calculate initial phases. Solvent flattening then resulted in a map of excellent quality (FOM = 0.58) into which >95% of the polypeptide chain was then built automatically. Repeated cycles of rebuilding in COOT (33, 34) and refinement with PHENIX (30) resulted in a structure with excellent geometry and R-factors. Mutant structures were determined by molecular replacement using the wt structure as the search model. Data and model statistics are given in Tables 1 and 2, respectively. The surface electrostatic potential shown in Figure 1 was calculated using the APBS (35) plugin for PyMOL (Schrödinger) and PDB2PQR (36). Parameters for the heme and calcium ions for use with APBS were adapted from the PARSE force field (37).

### Elemental analysis

ICP-OES was performed by the *Institut für Geowissenschaften*, Heidelberg University. Three 1 ml samples of 500 μM as-isolated, C-terminally His-tagged Kustd1711 in 25 mM Hepes–NaOH (pH 7.5) were investigated using an Agilent 720 ICP-OES instrument.

### UV–Vis spectroscopy

The protein was diluted in 25 mM Hepes, 25 mM KCl, pH 7.5, to an $A_{550\;nm}^{1\;cm}$ of ∼0.5, and spectra measured immediately in a 1 cm path length quartz cuvette (Hellma GmbH) using a Jasco V-760 spectrophotometer (Jasco GmbH). A reduced spectrum was obtained by measuring spectra after addition of TCEP to 2.5 mM from a 500 mM stock solution adjusted to pH 7.0. To oxidize the protein, 50 μM potassium ferricyanide was added, and spectra were measured using buffer with 50 μM potassium ferricyanide as blank because of the absorption of ferricyanide in the region of interest of the spectrum.

### Spectroelectrochemical characterization

Spectropotentiometric measurements were performed at room temperature as described previously (11, 38). Briefly, a custom-built OTTLE cell was used (Fig. S1), connected to a Keithley model 2450 source measure unit (Tektronix, Inc) employed as a potentiostat. A gold mesh was modified by incubation in 20 mM 4,4′-dithiodipyridine in 160 mM Tris–HCl, pH 8.0 and 20% (v/v) ethanol and used as the working electrode. About 18 μl of protein solution in 100 mM KCl, 10 mM Mops–KOH (pH 7.0) was mixed with 2 μl of a redox mediator mix (containing 1 mM each of potassium ferricyanide, *p*-benzoquinone, 2,5-dimethyl-*p*-benzoquinone, 1,2-naphtoquinone, phenazine methosulfate, 1,4-napthoquinone, phenazine ethosulfate, 5-hydroxy-1,4-napthoquinone, 2-methyl-1,4-napthoquinone, 2,5-dihydroxy-*p*-benzoquinone, 2-hydroxy-1,4-napthoquinone, anthraquinone, sodium anthraquinone-2-sulphonate, benzyl viologen, and methyl viologen) and placed in the cell. As required, 10 mM calcium acetate was added or the protein was treated with 100 mM EGTA on ice for 30 min, after which the buffer was exchanged to the spectroelectrochemistry buffer (see aforementioned) by repeated ultrafiltration. After sealing with parafilm, the cell was placed in a Jasco V-760 spectrophotometer (Jasco GmbH). Samples were first prepoised at −600 mV for 10 min and then oxidized to +400 mV and reduced back again to −600 mV, in steps of 50 mV. UV–Vis spectra were recorded at wavelengths from 350 to 700 nm (90 s/spectrum at 400 nm/min). The raw spectra were processed using the Jasco32 software and baseline corrected by setting the absorption at 700 nm to zero. The measurements from three individual series with freshly prepared solutions were averaged. In order to determine the midpoint potentials, the averaged absorbance at 417 nm (Soret band) as a function of potential was fitted using custom-written Python scripts to a multitransition Nernstian function:

$$Y=A-A_{ox}=\sum_{i}\frac{a_{i}}{e^{\left(\frac{zF}{RT}\left(E-E_{m,i}\right)\right)}+1}$$

where *A* is the absorbance, *A*_*ox*_ the absorbance of the fully oxidized state normalized to 1.0, $a_{i}$ the amplitude of the *i*-th transition, *E* the potential, $E_{m,i}$ the midpoint potential of the *i*-th transition, and *z* the number of electrons (*n* = 1). The Faraday constant F was taken to be 96,485.34 J V^−1^ mol^−1^, the temperature T was 293 K, and the ideal gas constant R was taken to be 8.3145 J mol^−1^ K^−1^. The potential was corrected against the SHE by measuring the voltage between the Ag–AgCl patch in a drop of 10 mM Mops–KOH (pH 7.0), 100 mM KCl, and a commercial Ag–AgCl–4 M KCl reference electrode (Pine Research Instrumentation; E° = +200 mV *versus* SHE).

### Theoretical prediction of redox potentials and affinities

The protein structure was prepared using the program CHARMM (39) with the CHARMM36 (40) force field parameters. The mutations Gln74Leu–Leu209Gln were introduced using Coot (33, 34). Missing hydrogen atoms were added with CHARMM using HBUILD and subsequently minimized, while all nonhydrogen atoms were kept fixed. The continuum electrostatic calculations were based on the Poisson–Boltzmann continuum electrostatic model (22). In all calculations, the dielectric permittivity was set to 4 for the protein and to 80 for the solvent using a probe sphere radius of 1.4 Å. The ionic strength was set to 0.1 M, and the ionic radius was set to 2.0 Å. The temperature was set to 300 K. Redox potentials and binding energies were calculated using MEAD (41) and GMCT (42). Atomic partial charges for the standard amino acids were taken from the CHARMM36 force field (40). To calculate the atomic partial charges for the heme center, coordinates of the heme with its two ligands retrieved from the structure of horse cytochrome *c* with the PDB code 6K9I (43). The geometry of the center in the oxidized and reduced states was optimized using the DFT functional B3LYP and the basis set def2-TZVP applying the program ORCA (44). The total spin was set to S = 1/2 and S = 0 for the oxidized and reduced state, respectively. Finally, the atomic charges for the model compound were calculated using CHELPG (45) within ORCA. The model redox potential was adjusted to obtain the experimental redox potential of 250 mV for horse cytochrome *c* (46) using the PDB structure 6K9I and electrostatic calculations with the same settings as aforementioned. The model binding energy of the calcium ion was set to zero. Thus, since the two ion binding sites have the same coordination geometry, relative binding energies can be calculated.

### Bioinformatics

Custom-written Python scripts using the PyPDB module (47) were used to download PDB files from the PDB (48) containing at least one heme (HEM or HEC) and one calcium ion. All distances between the calcium ions and the heme iron atoms were calculated and stored for analysis.

### Differential scanning fluorimetry

DSF measurements were performed with a Prometheus NT.48 (NanoTemper Technologies) at 100% excitation energy, using a temperature gradient of 20 to 95°C at 1 K min^−1^. Protein samples (around 10 μl in microcapillaries) were measured in triplicates at concentrations of 1 mg ml^−1^ in the same buffer that was used for the electrochemical measurements (100 mM KCl, 10 mM Mops–KOH [pH 7.0]), with or without 10 mM potassium ferricyanide, 10 mM calcium chloride, or 10 mM EGTA, as required. The “Ox/EGTA” sample was first treated with 100 mM EGTA, 10 mM ferricyanide in electrochemistry buffer for 30 min on ice, after which the buffer was exchanged by ultrafiltration to electrochemistry buffer with 10 mM EGTA for the DSF measurement, for consistency with the electrochemical measurements.

### Estimation of calcium dissociation constant

The protein's apparent *K*_*d*_ for Ca^2+^ was measured using the CalBryte-590 fluorescent calcium sensor (Biomol GmbH), because the emission and excitation wavelengths of this dye do not coincide with spectral features of the protein (Fig. 4*B*). First, a calibration series containing 0 to 5 mM total calcium in 5 mM EGTA was made using the calcium calibration kit from ThermoFisher Scientific in assay buffer (20 mM Mops, pH 7.0, 100 mM KCl) with 20 μM CalBryte-590. Fluorescence was measured from 150 μl samples (prepared in triplicates) in a black 96-well plate (Corning, ThermoFisher Scientific) using a Spark multimode plate reader (Tecan Deutschland GmbH), exciting at 570 nm and measuring the emission at 610 nm, where the protein itself shows little absorption (Fig. 4*A*); 30 flashes, an integration time of 40 μs and a gain setting of 30 were used, and a 20 nm bandwidth was selected for both excitation and emission. Using the cubic equation (49) and assuming *K*_*d*_ values for calcium of 1.0 × 10^−7^ mol l^−1^ for EGTA (50) and of 1.4 × 10^−6^ mol l^−1^for CalBryte-590 (according to the manufacturer), the concentrations of the Ca^2+^.dye complex could be calculated as varying between 0 and 18 μM. A second-order polynomial was then used to calibrate the correspondence between fluorescence signal and Ca^2+^.dye complex concentration.

Next, a series of samples was prepared with protein concentrations varying from 0 to 3.5 μM, from a 0.5 mM protein stock as determined from the absorption at 550 nm, assuming an extinction coefficient of 62 l mmol^−1^ cm^−1^ as determined using the pyridine hemochrome assay (51). The protein was diluted in assay buffer containing 20 μM CalBryte-590 (in triplicates), and the fluorescence was measured as before both immediately and after 1, 2, and 3 h. No significant change in signal was detected over 3 h. Using the calibration curve determined earlier, a background of 2.4 ± 0.2 μM Ca^2+^ was detected in protein-free samples. Plotting the observed Ca.dye complex concentration as a function of protein concentration revealed an approximately linear trend. Using the cubic equation and assuming a dissociation constant for calcium of 1.4 × 10^−6^ mol l^−1^ for CalBryte-590, a family of curves was calculated for dissociation constants for calcium of the protein varying between 1 nM and 10 μM (taking the background calcium concentration into account, and assuming 1.6 calcium ions per protein molecule as per the ICP-OES results). These curves were compared with the observed data to estimate an upper limit for the apparent *K*_*d*_.

## Data availability

Crystal structures and diffraction data were deposited in the PDB (48) under accession codes 7ZS0, 9FBK, 7ZS1, and 7ZS2 for the wt protein cryoprotected with glycerol, the wt protein without glycerol, the M292C mutant, and the M292H mutant, respectively. All other data are available from the authors on request.

## Supporting information

This article contains supporting information.

## Conflict of interest

The authors declare that they have no conflicts of interest with the contents of this article.

## Supporting information

Supplemental Figs. S1 and S2

Heme_Calcium_Distances
