# Supplementary material for: Redox potential tuning by calcium ions in a novel c-type cytochrome from an anammox organism[image]
Source: J Biol Chem. 2024 Dec 13;301(2):108082. doi: 10.1016/j.jbc.2024.108082 (PMC11791136; doi:10.1016/j.jbc.2024.108082)
Supplement: Supplemental Figs. S1 and S2 [file mmc1.pdf]

SUPPORTING INFORMATION

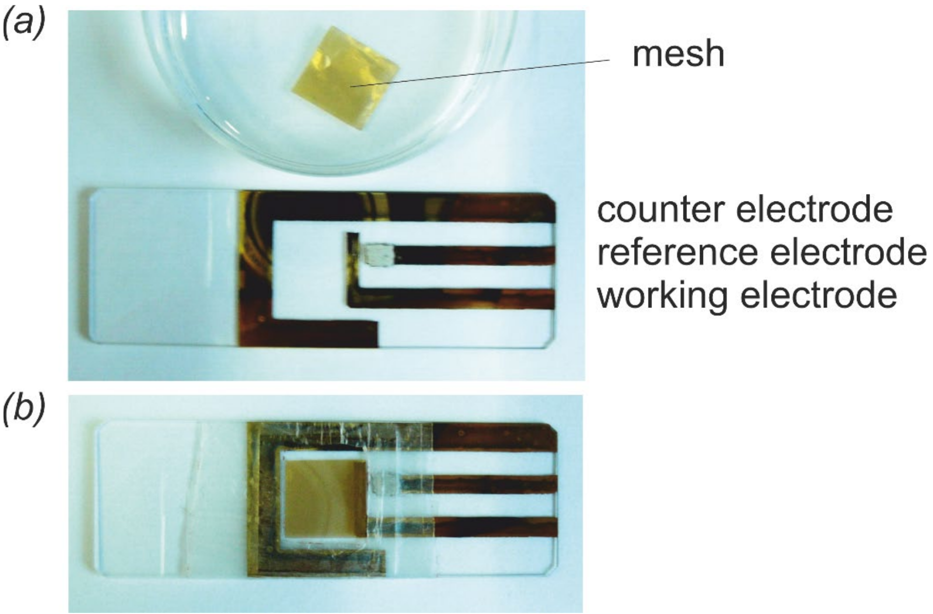

Figure S1. OTTLE cell design. (a.) Photograph of the microscope slide-based OTTLE cell. Traces for the counter-, working- and reference electrodes are prepared with "Glanzgold" gold paint, and a patch of Ag/AgCl ink forms the reference electrode. The top of the picture shows a 10 µm thick gold mesh kept in its derivatization solution. (b.) photograph of the assembled cell, filled with 20 µl of sample, closed with a 24×24 mm coverslip and sealed with parafilm. The cell is then placed in the beam path of a UV/Vis spectrophotometer.

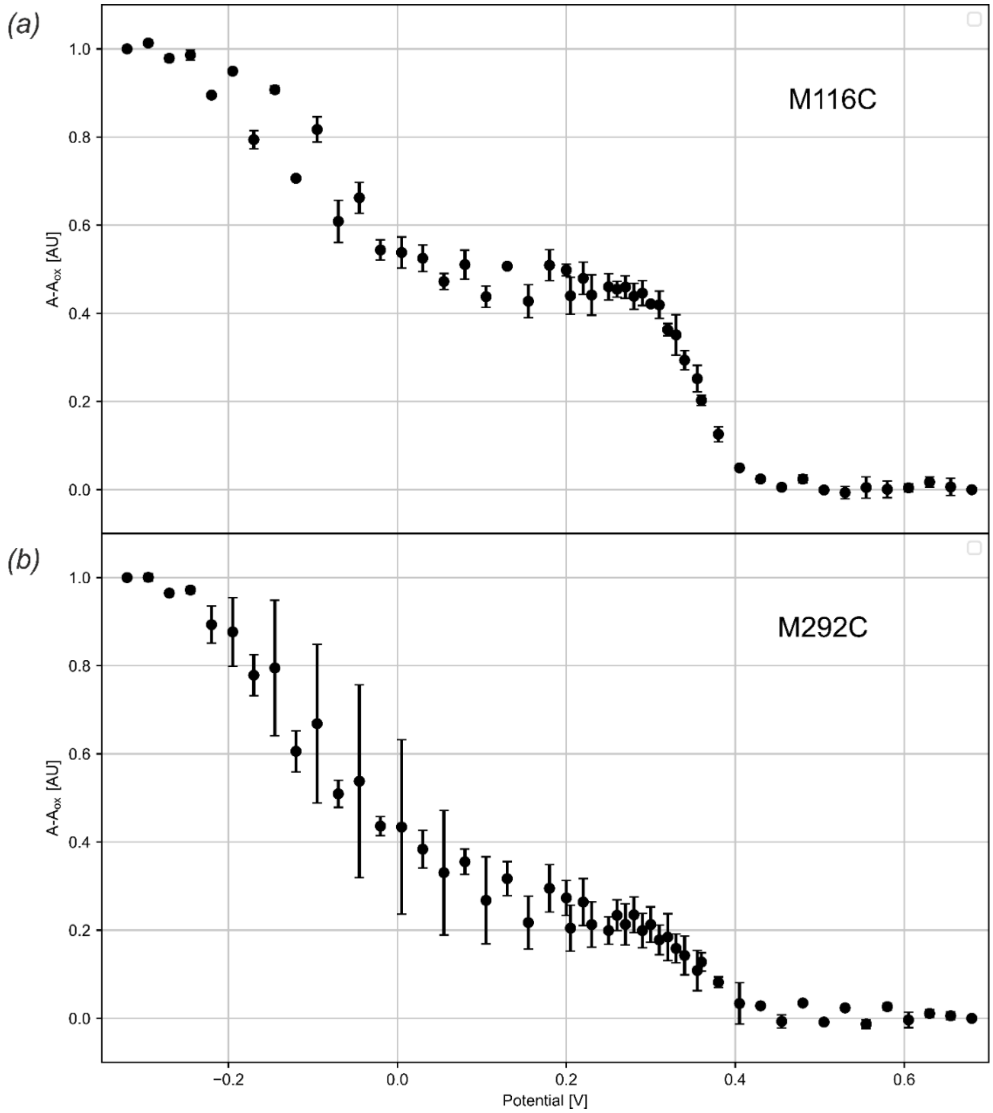

Figure S2. Spectroelectrochemical results obtained with the M→C mutants of Kustd1711. The hysteresis and poorly defined transitions at low potentials preclude fitting with a Nernstian equation. (a) M116C mutant. (b) M292C mutant. The potential (x-axis) is indicated in volts vs. standard hydrogen electrode.
